# Pandemic (H1N1) 2009 and HIV Co-infection

**DOI:** 10.3201/eid1702.101775

**Published:** 2011-02

**Authors:** David T. Kuhar, David K. Henderson

**Affiliations:** Author affiliations: Centers for Disease Control and Prevention, Atlanta, Georgia, USA (D.T. Kuhar);; National Institutes of Health, Bethesda, Maryland, USA (D.K. Henderson)

**Keywords:** Influenza, viruses, co-infection, pandemic (H1N1) 2009, HIV, letter

**To The Editor**: Barchi et al. report a case of simultaneous pandemic (H1N1) 2009 influenza and HIV infection (*1*). We agree with the authors’ conclusion that during influenza epidemics, consideration of alternative diagnoses, such as acute HIV infection, remains essential for patients who seek treatment for severe influenza-like illnesses. However, from our perspective, several points from this letter need additional clarification.

First, we recommend that the authors clarify whether the positive HIV test results reported were for the hospitalized patient (as we suspect) or for the nurse who was exposed to the patient’s urine. Occupationally acquired HIV infection in a health care provider after an ocular splash with urine has, to our knowledge, never been reported and, if these test results are for the worker, would represent a novel source of transmission. Precision with respect to the source of these samples and results is critical to reader understanding.

Additionally, the reported negative Western blot results demonstrated p24 and p41 bands; this test result would be considered positive by Centers for Disease Control and Prevention–endorsed interpretive criteria (i.e., Western blot positivity equates to presence of any 2 of the following 3 bands: p24, p41, and gp120/160) (*2*). Thus, the negative Western blot result interpretation, even if caused by different local interpretive criteria, deserves further explanation.

Finally, diagnosing acute HIV infection can be challenging. Although the elevated initial CD4 lymphocyte percentage and viral load are suggestive of recent HIV infection (*3*), the ELISA result was positive. Do the authors have access to a prior HIV test result that may shed further light on the chronicity of HIV infection? The hepatitis C infection in this patient was also diagnosed relatively recently. Co-infection with HIV and hepatitis C virus may alter the course of both infections and may have contributed to the severity of this patient’s illness (*4*).
