# Supplementary material for: Pandemic (H1N1) 2009 and HIV Co-infection
Source: Emerg Infect Dis. 2011 Feb;17(2):328. doi: 10.3201/eid1702.101775 (PMC3298381; doi:10.3201/eid1702.101775)
Supplement: Related texts

# Reply

**Authors:** Enrico Barchi

**Affiliations:** Author affiliation: Ospedale Santa Maria Nuova, Reggio Emilia, Italy

**Keywords:** Keywords: Viruses, influenza, HIV, pandemic (H1N1) 2009, HIV seroconversion, co-infection, letter

**In Response:** In reply to Kuhar and Henderson (*1*), the positive HIV test result reported was for the patient. The nurse who was exposed to the patient’s urine is currently HIV negative 12 months after exposure. With regard to the negative result for the Western blot, part of the original sentence was accidentally changed in the published letter (*2*); the correct information is “confirmatory Western blot test results were negative on days 5 and 15, and showed the p24 and p41 bands on day 23.” Finally, a prior HIV test, which had been performed for the patient 8 months earlier, showed a negative result.

## References

[1] Kuhar DT, Henderson DK. Pandemic (H1N1) 2009 and HIV co-infection [letter]. Emerg Infect Dis. 2011;17:328.2129162710.3201/eid1702.101775PMC3298381

[2] Barchi E, Prati F, Parmeggiani M, Tanzi ML. Pandemic (H1N1) 2009 and HIV co-infection [letter]. Emerg Infect Dis. 2010;16:1643–4.2087530410.3201/eid1610.100341PMC3294392
